# Lime Inhalations in Pseudo-Membranous Croup

**Published:** 1867-06

**Authors:** Benjamin B. Wilson

**Affiliations:** Late Surgeon and Lieut. Colonel, U. S. V. of Philadelphia


### SELECTIONS.

---

*Lime Inhalations in Pseudo-Membranous Croup.* By BENJAMIN B. WILSON, Late Surgeon and Lieut. Colonel, U. S. V. of Philadelphia.

I have recently attended a case of pseudo-membranous croup, in which I prescribed lime water inhalations, and in which the curative effects of this remedy were most marked and decided.

When first called to my patient, a child about two years of age, the disease had existed for more than forty-eight hours; though up to this time, little alarm had been excited in the minds ot the parents from the apparent slow progress and insignificant character of the affection. During most of the time, the child had kept upon her feet about the house during the day; and some fever, with difficulty of breathing and an occasional cough were the most prominent symptoms aggravated, of course, during the night, but without any distressing complications. A more decided exacerbation, however, had preceded my being sent for, and I found her with all the symptoms of the disease fairly and strongly developed. There was a high febrile action with very considerable dyspnœa; the voice was almost extinct, a hoarse dry whisper being the only result of any effort to articulate, and there was present the occasional (though not very frequent) loud, barking, ringing cough, characteristic of the disease.

Believing that the time for obtaining any remedial effect from venesection had passed, the child was immediately placed in a hot mustard bath for twenty minutes, and after being freely and repeatedly vomited and subjected to the action of counter-irritants in the shape of strong turpentine stupes to the neck and breast, the following prescription was ordered to be administered every two hours: of calomel ¼ gr., turpeth mineral, ¼ gr., ipecacuanha ¼ gr., sal ammoniac, one grain. This prescription was given during the entire day and evening, with the effect of keeping up constant nausea and occasional emesis, and a moderate purgation during the latter part of the evening, without however, producing any abatement of the threatning symptoms, which were, in fact, becoming every hour graver in their character, so that the case now assumed a threatening aspect.

In the evening, after a second warm mustard bath, the lime water inhalations were commenced, the other treatment being continued as before. A vapor bath was extemporized by throwing a large blanket over the child's head and shoulders as well as the sofa upon which it reclined and including also within its circumference a pitcher in which a small lump of quick lime was being rapidly slaked by means of boiling water. In this way the air surrounding the child's body, as well as that respired, was highly charged with the vapor of lime water. This process was repeated every hour, and sometimes every half hour, when the breathing seemed more than usually hurried and difficult. The immediate effect of this vapor bath upon the patient seemed to be soothing, the most urgent dyspnœa being relieved, and the little sufferer almost always bocoming quieter and falling into a light sleep under its use. The constant repetition of the inhalations, seemed to check the steady progress of the symptoms from bad to worse, and to mitigate in a very considerable degree the most distressing and dangerous one, that of impeded respiration. This treatment, substantially, was kept up during the whole night, the day following, and the succeeding night; there being, it was thought, a constant though gradual, and slow improvement during that time. On the next morning, forty-eight hours after I took charge of the case, and ninety-six from the inception of the disease, the symptoms gave way; the cough suddenly becoming loose and catarrhal in its character, and the breathing free and uninterrupted; the case entering at once upon convalescense. The recovery, under the use of expectorants, was prompt and rapid, and with but a slight trace of the usual bronchial inflammation, which almost invariably is a disorder consequent upon severe cases of croup. The happy result in this case, under circumstances evidently so desperate, could be attributed only to the means employed in addition to the ordinary treatment; for one has only to be familiar with the disease, to know how powerless is the most approved treatment ordinarily, when directed against it.

I have previously used lime water inhalations in four cases of croup, in which the pseudo-membranous element was supposed to exist to a greater or less degree. They all recovered, and in each of them the remedy was considered to have been not without a beneficial effect, though to what extent was not and could not be determined. Two of these cases were also prescribed for by practitioners among the most eminent in Philadelphia, and were considered by them very serious, not to say hopeless cases. Treatment recognized as most efficient and proper for such cases was unintermittedly kept up, and how much credit, if any, of the cure could be fairly claimed by the inhalations, at the present at least admits of discussion. Sufficient apparent benefit was observed, however, at every trial, to warrant a further perseverance in its use.

The remedy may be administered by slaking lime in an ordinary inhaler, with a flexible tube and mouth-piece attached, or by a temporary vapor bath in the manner above described. The latter I prefer for several reasons, which become apparent in practice. The vapor as usually given off is at too high a temperature to be respired, unless diluted with a certain amount of atmospheric air at a lower temperature; and besides, the little sufferer, parched with fever and gasping for breath, is too much worried and frightened, even if naturally manageable, to be induced to breathe systematically and carefully through the mouth-piece of an inhaler. The vapor bath avoids all this, and can conveniently be applied during the intervals of temporary repose or sleep, and has the additional advantage of producing a profuse perspiration, which is of itself grateful and relaxing in no inconsiderable degree.

I refrain from theorizing upon the therapeutic action of this remedy, and from guessing how or why it produces its curative effects a tolerably moist atmosphere is confessedly the proper one for every case of croup, and doubtless the vapor of water alone in a certain proportion in the inspired air, increases the moisture of the respiratory mucous membranes and promotes secretion therefrom. Whether the hydrated lime with which the vapor is highly charged, has a specific action in dissolving, softening or loosening false membrane, or in promoting secretions from the mucous membrane of the larynx or trachea, is yet to be determined.

I, however, respectfully and earnestly call the attention of the profession to this remedy for the following reasons:

That pseudo-membranous croup is a very grave disorder, but little amenable to any treatmeut hitherto used when fully established; and even when seen and treated at the outset, often setting at defiance the most approved plans of practice which have been recommended up to the present time.

That it can be used in addition to and without interfering with any general treatment which may be judged appropriate in any particular case, and it may, under doubtful circumstances turn the scale in favor of life and recovery.

That it does not seem injurious in any respect; so far as used, no unpleasant result seems to have followed it, either immediately or remotely.

It is apparently soothing and most comforting to the patient, relieving the distressing symptoms, giving ease and rest, and disposing the little sufferer to sleep.

I have not thought it worth while to try it in catarrhal or purely spasmodic forms of the disease. These varieties as a rule yield promptly to appropriate active treatment. In no disease probably is the effects of medication more obviously apparent than in spasmodic croup; where, upon the exhibition of an emetic, the patient passes at once from a state of great seeming danger to one of almost entire relief, comfort, and safety; presenting in this respect the most marked contrast to the pseudo-membranous form of the disease.—*Med. and Surg. Reporter.*

